# Detection of Retinal Neurovascular Coupling During Light Adaptation Using Optical Coherence Tomography Angiography: A Pilot Study

**DOI:** 10.3390/life16071109

**Published:** 2026-07-03

**Authors:** Ágnes Élő, Lilla István, András Attila Horváth, Krisztina Horváth, Tamás Ódor, Tamás Andorfi, Zoltán Zsolt Nagy, Illés Kovács

**Affiliations:** 1 Department of Ophthalmology, Semmelweis University, H-1085 Budapest, Hungary; elo.agnes@semmelweis.hu (Á.É.); istvan.lilla@semmelweis.hu (L.I.); nagy.zoltan.zsolt@semmelweis.hu (Z.Z.N.); 2 Neurocognitive Research Center, Nyírő Gyula National Institute of Psychiatry, and Addictology, H-1145 Budapest, Hungary; horvathandras@nyiro-opai.hu; 3 Department of Anatomy, Histology and Embryology, Semmelweis University, H-1094 Budapest, Hungary; 4 Faculty of Medicine, Universidade de Lisboa, 1649-028 Lisbon, Portugal; 5 Institute of Cognitive Neuroscience and Psychology, Research Centre for Natural Sciences, Hungarian Research Network, H-1117 Budapest, Hungary; 6 Faculty of Medicine, Semmelweis University, H-1085 Budapest, Hungary; horvath.kriszta12@gmail.com (K.H.); vermokus2000@gmail.com (T.Ó.); 7 Department of Clinical Ophthalmology, Faculty of Health Sciences, Semmelweis University, H-1085 Budapest, Hungary; andorfitamas@gmail.com; 8 Department of Ophthalmology, Weill Cornell Medicine, New York, NY 10021, USA

**Keywords:** optical coherence tomography angiography, neurovascular coupling, retinal blood flow

## Abstract

**Background:** Neurovascular coupling (NVC) is a fundamental mechanism that dynamically matches retinal blood flow to neuronal metabolic demand. While dynamic vessel analysis (DVA) has been established for assessing NVC through flicker-light stimulation, the potential of optical coherence tomography angiography (OCTA) to detect NVC during physiological stimuli, such as dark-to-light adaptation, remains unexplored. **Methods:** In this prospective cross-sectional study, OCTA imaging was performed in both eyes of 22 healthy participants under dark-adapted (scotopic) and light-adapted (photopic) conditions. Each condition was measured three times consecutively. Macular and peripapillary vessel density (VD) were quantified. **Results:** After adjustment for measurement order and scan quality, light adaptation significantly increased peripapillary small VD (Δ = +1.30%, *p* = 0.046; 95% CI: 0.03–2.56%). Peripapillary all VD demonstrated a similar trend but remained borderline significant (Δ = +1.19%, *p* = 0.069). In contrast, macular VD showed no significant association with light adaptation (Δ = −0.91%, *p* = 0.11; 95% CI: −2.02 to 0.21%), but was significantly affected by scan quality (Δ = 1.62%, *p* < 0.001, 95% CI: 1.23–2.02%). **Conclusions:** In healthy older adults, OCTA detected an increase in peripapillary VD associated with dark-to-light adaptation, reflecting retinal vascular reactivity consistent with neurovascular coupling. The pronounced influence of scan quality and measurement order underscores their importance as critical confounding factors that must be carefully controlled in functional and longitudinal OCTA studies. Together, these findings highlight OCTA’s promise as a non-invasive tool for assessing retinal neurovascular reactivity, while emphasizing the need for scan quality standardization and order correction to ensure reliable interpretation.

## 1. Introduction

The retina, as an extension of the central nervous system (CNS), shares fundamental anatomical, physiological, and embryological characteristics with the brain [1,2]. Both structures derive from pluripotent neuroectodermal cells of the diencephalon and display similar microvascular architectures [3]. The retina is among the most metabolically active tissues in the human body, with oxygen consumption rates comparable to those of the brain, necessitating precise regulation of blood flow to meet dynamic metabolic demands [2,4,5].

The neurovascular unit (NVU) describes the functional and anatomical interactions among neurons, glial cells (primarily Müller cells and astrocytes), vascular endothelial cells, and pericytes [6,7,8]. This integrated system maintains a balance between local neural activity and perfusion through neurovascular coupling (NVC), also referred to as functional hyperemia [9,10]. NVC ensures that neuronal activation promptly induces vasodilation to increase local oxygen and nutrient supply. Within the retina, NVU components are distributed across three capillary plexuses—superficial, intermediate, and deep—each supporting different retinal layers [11]. Although various signaling cascades participate in NVC [6,7,12,13,14,15,16], three primary pathways are recognized: (1) nitric oxide (NO) released from interneurons and ganglion cells induces smooth muscle relaxation via cyclic GMP elevation [17,18,19,20]; (2) purinergic signaling, in which ATP from Müller cells activates glial and vascular receptors, triggering vasoactive metabolite release such as prostaglandin E2 and epoxyeicosatrienoic acids [9,21,22]; and (3) pericyte control, whereby contractile pericytes modulate capillary diameter in response to vasoactive mediators [10,23,24,25,26,27,28]. Collectively, these processes ensure rapid microvascular adjustment to neuronal activity.

Adaptation to changing illumination provides a natural model for studying NVC [29,30]. Under dark adaptation (scotopic conditions), rod photoreceptors dominate retinal function and require continuous metabolic support. In contrast, light adaptation (photopic conditions) activates cone-mediated pathways and inner retinal circuits, increasing metabolic demand [13,31,32,33]. Multifocal electroretinography (mfERG) demonstrates functional alterations in photoreceptor and bipolar cell responses during these transitions [34,35]. Impaired dark adaptation correlates with microvascular dysfunction in diseases such as diabetic retinopathy, where reduced perfusion accompanies impaired neuronal responses [36,37,38]. Hence, the physiological shift between scotopic and photopic conditions can serve as a natural NVC stimulus without the need for external flicker.

Dynamic Vessel Analysis (DVA) is an established reference method for assessing flicker-induced NVC, utilizing flicker-light stimulation (typically 12.5 Hz) to induce NVC-associated changes in vessel diameter [30,39]. DVA provides reproducible functional measures—arterial dilation, venous dilation, and baseline-corrected flicker response amplitude—and has revealed early microvascular impairment in diabetes, hypertension, and carotid stenosis [40,41,42]. Although DVA is an established method for assessing flicker-induced NVC, it has important limitations: it requires dedicated, specialized equipment that is not widely available, interrogates only the larger retinal arterioles and venules rather than the capillary bed, depends on active flicker stimulation and sustained patient cooperation, and is sensitive to media opacity and fixation instability. Optical Coherence Tomography Angiography (OCTA) has transformed non-invasive retinal vascular imaging by providing high-resolution views of the microcirculation without dye injection [43]. OCTA offers complementary advantages—wide clinical availability, rapid dye-free acquisition, depth-resolved capillary-level quantification, and integration into routine imaging—but samples a different segment of the vascular tree (the capillary plexus rather than large vessels). The technique is based on split-spectrum amplitude-decorrelation angiography (SSADA), detecting red blood cell motion between sequential scans [43,44]. OCTA enables separate visualization of the macular and peripapillary perfusion, facilitating assessment of vessel density (VD) across distinct retinal layers [45]. OCTA demonstrates excellent reproducibility and has become integral in evaluating microvascular alterations in numerous retinal and systemic diseases [46,47]. Quantitative OCTA metrics such as VD, perfusion density, and foveal avascular zone (FAZ) area have shown diagnostic and prognostic significance in diabetic retinopathy, glaucoma, and age-related macular degeneration [48,49,50,51]. Beyond ophthalmology, OCTA has revealed retinal microvascular deficits in neurodegenerative and cerebrovascular conditions, including Alzheimer’s and Parkinson’s diseases, underscoring its potential as a biomarker of CNS vascular integrity [52,53,54,55,56,57,58,59,60,61].

Nevertheless, several methodological factors constrain OCTA interpretation. Image quality depends strongly on media transparency, fixation stability, and signal strength [62,63,64]. Variations in scan quality can introduce systematic biases in vascular density measurements [65,66]. Moreover, OCTA primarily captures static structural information rather than dynamic vascular reactivity, with temporal resolution insufficient to track rapid perfusion changes [44]. Consequently, whether OCTA can detect the subtle hemodynamic shifts characteristic of physiological NVC remains uncertain. Previous research has established OCTA sensitivity to pathological alterations—such as capillary nonperfusion and neovascularization—but its ability to measure transient activity-driven blood flow remains unproven [67]. Given the widespread accessibility of OCTA, its non-invasive nature, and its ability to provide layer-specific quantitative data, investigating its capacity to assess functional perfusion changes holds significant clinical potential [68]. If validated for NVC analysis, OCTA could serve as a complementary technique to DVA, offering a practical, time-efficient, and widely deployable biomarker of neurovascular integrity [69,70]. The transition from dark to light adaptation provides a physiological paradigm that naturally modulates retinal metabolism and blood flow [71,72]. During the dark-to-light transition, cone-mediated pathways and inner-retinal circuitry are activated, increasing inner-retinal metabolic demand. Through NVC—mediated by nitric oxide, glial purinergic signaling, and pericyte responses—this increased demand triggers functional hyperemia, i.e., capillary vasodilation and recruitment that enlarge the perfused capillary area. Because OCTA-derived VD quantifies the proportion of tissue area occupied by flowing blood, such capillary recruitment and dilation are expected to raise measured VD even without resolving individual vessel-diameter changes. OCTA thus detects an area- or recruitment-based surrogate of the neurovascular response rather than the vessel-diameter changes measured by DVA, which is why the expected OCTA signal is comparatively small and may be region-specific. Recent work has begun to image illumination-driven retinal vascular responses directly: single-capillary-resolution functional OCTA has resolved retinal functional hyperemia [73], and dark-to-light adaptation OCTA has revealed reversed vascular reactivity associated with light adaptation, consistent with NVC as an early abnormality in diabetes [69]. In healthy eyes, light conditions have been shown to influence peripapillary OCTA parameters, with a significant increase in peripapillary VD from dark-to-light adaptation [74]. Demonstrating that OCTA can detect such neurovascular adjustments would establish the method as a convenient tool for functional vascular assessment during routine imaging [75].

The purpose of this study was to determine whether OCTA can detect retinal VD changes associated with vascular reactivity due to light adaptation, consistent with NVC during dark-to-light adaptation, and to evaluate the influence of scan quality on detecting NVC-related variations and the overall feasibility of OCTA as a functional imaging tool for retinal NVC assessment.

## 2. Materials and Methods

This prospective, observational, cross-sectional study was conducted at the Department of Ophthalmology, Semmelweis University, Budapest, Hungary. The study protocol adhered to the tenets of the Declaration of Helsinki and received approval from the Institutional Review Board of Semmelweis University (approval number: 144/2023). Written informed consent was obtained from all participants prior to enrollment.

### 2.1. Participants

Twenty-two healthy participants with no history of ocular or systemic disease were recruited. Inclusion criteria were: (1) age 50–75 years, (2) best-corrected visual acuity ≥ 20/25, (3) normal ophthalmological examination, (4) clear ocular media, and (5) ability to cooperate with imaging procedures. Exclusion criteria included: (1) any retinal pathology, (2) glaucoma or intraocular pressure > 21 mmHg, (3) refractive error > ±6 diopters, (4) history of ocular surgery, (5) systemic diseases affecting the vasculature (diabetes, hypertension, cardiovascular disease), (6) neurological disorders, and (7) current medication use affecting vascular tone. Participants were restricted to the 50–75-year age range to target the period of greatest interest for early neurovascular dysfunction while minimizing the confounding effects of age-related media opacities and reduced fixation stability on OCTA image quality. All participants underwent a comprehensive ophthalmological examination, including slit-lamp biomicroscopy, dilated fundus examination, intraocular pressure measurement, and assessment of best-corrected visual acuity.

### 2.2. OCTA Imaging Protocol

OCTA imaging was performed using the RTVue XR Avanti system with AngioVue software version 2017.1, phase 7 update (Optovue Inc., Fremont, CA, USA). This spectral-domain OCT device operates at a central wavelength of 840 nm with a scan speed of 70,000 A-scans per second. The system utilizes the split-spectrum amplitude-decorrelation angiography (SSADA) algorithm to derive flow information by detecting motion contrast from erythrocyte movement between sequential B-scans. Retinal layer segmentation and VD calculations were generated automatically using the AngioAnalytics software (version 2017.1, phase 7). For each acquisition, the scan quality (SQ) index—a unitless metric ranging from 0 to 10, with higher values indicating better image quality—was recorded; this index incorporates contributions from signal strength, motion artifacts, and focus quality.

### 2.3. Imaging Procedure

Participants underwent dark adaptation in a completely darkened room for 20 min prior to imaging, seated with eyes closed under ambient illumination <0.1 lux as verified by a calibrated light meter. After dark adaptation, macular OCTA imaging was performed using the minimal device illumination, acquiring three consecutive 3 × 3 mm macular and three 4.5 × 4.5 mm peripapillary scans from each eye. Subsequently, subjects were light-adapted by exposure to ambient room lighting (approximately 300 lux) for 10 min, followed by three consecutive 3 × 3 mm macular and three 4.5 × 4.5 mm peripapillary OCTA scans per eye under photopic conditions. All imaging was performed by a single experienced operator (L.I.) with the automated eye-tracking function activated to reduce motion-related variability. Only scans with scan quality (SQ) ≥ 5 and without significant motion artifacts, segmentation errors, or projection artifacts were included in the final analysis.

### 2.4. OCTA Image Analysis

Quantitative OCTA analysis was performed using the AngioAnalytics software, which automatically extracted vessel density parameters. The following parameters were analyzed: (1) macular VD, defined as the percentage of area occupied by perfused vessels within the entire 3 × 3 mm scan; (2) peripapillary small VD, representing the density of capillaries with a diameter <20 µm; and (3) peripapillary all VD, representing the total density of both small and larger peripapillary vessels. For each scan, the software-reported scan quality (SQ) was recorded for macular VD measurements, and peripapillary scan quality (SQP) was recorded for peripapillary VD metrics. All OCTA images underwent standardized quality control by two independent graders (L.I., K.H.), who were masked to the experimental condition. Images were evaluated for: (i) sufficient signal strength (SQ ≥ 5), (ii) correct automated segmentation, (iii) absence of motion artifacts (e.g., vessel doubling, horizontal or vertical white line artifacts), (iv) absence of prominent projection artifacts, and (v) absence of significant shadowing from media opacities. Image quality was uniformly high: every acquired scan met the predefined signal-quality threshold, and therefore, no scan required repetition or was excluded for inadequate quality. When segmentation errors were identified, they were manually corrected using the built-in segmentation editing tools before quantitative analysis.

### 2.5. Statistical Analysis

Based on previous studies reporting VD standard deviations of approximately 2–3% and assuming a minimum clinically meaningful difference of 0.5% between dark and light adaptation conditions, a sample size of 40 eyes (20 subjects) was calculated to provide 80% power at α = 0.05; the present study enrolled 22 subjects (44 eyes), exceeding this target. Data were analyzed using IBM SPSS Statistics for Windows (Version 31.0, Armonk, NY, USA: IBM Corp.) and Python 3.10 (Python Software Foundation, Wilmington, DE, USA). Continuous variables were expressed as mean ± SD or median (interquartile range, IQR) according to normality (Shapiro–Wilk test). Linear mixed-effects models with patient as a random intercept accounted for repeated measurements within eyes and bilateral correlation. Because the protocol fixed dark-adapted measurements to positions 1–6 and light-adapted to positions 7–12, a sequential position covariate (1–12) was added to control for fatigue or signal degradation, and four nested models were fitted per metric: M1 (unadjusted), M2 (+scan quality), M3 (+position), and M4 (+scan quality + position). A participant-level random intercept linked all repeated measurements from each subject—both eyes and their consecutive acquisitions—thereby modeling within- and between-eye correlation and the corresponding reduction in effective sample size. Model fit was compared using Akaike’s information criterion (AIC), Bayesian information criterion (BIC), and conditional R^2^ (Nakagawa–Schielzeth). Results are reported as β coefficients with 95% CI and *p*-values. Finally, to assess the robustness of the fully adjusted model (M4), a cluster bootstrap procedure was performed with 1000 patient-level resamples, and percentile 95% confidence intervals and significance rates were computed.

## 3. Results

Twenty-two healthy participants (44 eyes) were enrolled in the study. Baseline demographic and ocular characteristics are summarized in Table 1. All participants had normal visual acuity, normal intraocular pressure, and clear ocular media without evidence of cataract or vitreous opacities. Overall, 264 macular OCTA scans were acquired from 22 participants (3 consecutive 3 × 3 mm scans × 2 eyes × 2 conditions = 12 scans per participant; 132 under scotopic and 132 under photopic conditions).

**Figure 1:**
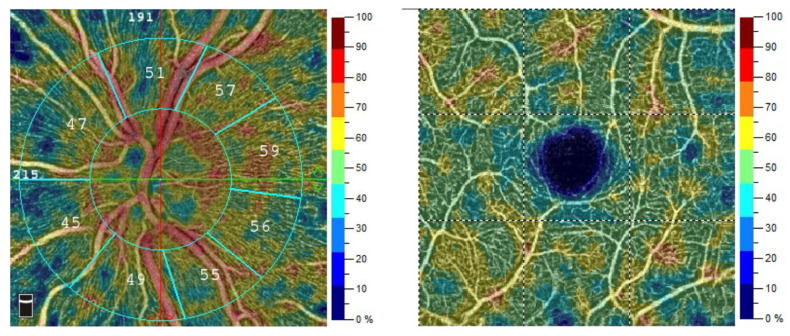
Representative OCTA images analyzed with AngioAnalytics software. (**Left**) Peripapillary scan, yielding peripapillary small VD (capillaries < 20 µm) and peripapillary all VD (small plus larger vessels). (**Right**) Macular scan (3 × 3 mm), yielding macular VD, the percentage of area occupied by perfused vessels. In both panels, the color scale adjacent to each image encodes local VD, ranging from low (blue) to high (red), as indicated by the 0–100% color bar.

Image quality was comparable across adaptation conditions. Macular scan quality averaged 7.78 ± 0.90 under dark adaptation and 7.70 ± 0.84 under light adaptation (overall 7.74 ± 0.87; range 6–9), while peripapillary scan quality averaged 8.26 ± 0.82 under dark adaptation and 8.13 ± 0.88 under light adaptation (overall 8.19 ± 0.85; range 6–10). Scan quality did not differ significantly between the two conditions for either region (macular *p* = 0.51; peripapillary *p* = 0.23), confirming that the adaptation-related differences in VD were not driven by systematic differences in image quality. Representative peripapillary and macular OCTA scans, with their VD color maps, are presented in Figure 1, illustrating the parameters extracted for quantitative analysis.

Because the fixed examination sequence confounded the dark-to-light comparison, sequential measurement position (1–12) was entered as an additional covariate (Table 2). For macular VD, measurement order was not a significant predictor (β = 0.11 per position; *p* = 0.18), and the light-adaptation effect remained non-significant after adjustment for both SQ and order (β = −0.91; *p* = 0.11; conditional R^2^ = 0.57). By contrast, for peripapillary small VD, sequential position was a significant negative predictor (β = −0.23 per position; *p* = 0.021), indicating a progressive decline in measured density across the 12-measurement sequence. After controlling for this order effect, the light-adaptation coefficient reached statistical significance (Δ = +1.30%; *p* = 0.046; 95% CI: 0.03–2.56; conditional R^2^ = 0.58). A similar pattern was observed for peripapillary all VD, where measurement order was again significant (β = −0.22 per position; *p* = 0.02), and the light-adaptation effect approached significance (Δ = +1.19%; *p* = 0.069; conditional R^2^ = 0.57). Including sequential position markedly improved model fit for the peripapillary metrics but not for macular VD, identifying progressive intra-session signal degradation—likely from fatigue, reduced fixation stability, or tear-film changes—as a systematic influence that lowered later measurements and masked the underlying peripapillary adaptation effect. After accounting for both scan quality and measurement order in the fully adjusted model (M4), a clear and significant positive light-adaptation effect emerged in the peripapillary region, indicating that rigorous confounder control was necessary to reveal a genuine response that unadjusted analyses had obscured. Because adaptation condition and measurement order are confounded by the fixed examination sequence, this pattern is strongly consistent with a physiological adaptation effect, though the design does not allow it to be proven definitively. Peripapillary scan quality (SQP) was not a significant predictor in any model (all *p* > 0.39), confirming that the order effect captured variance not explained by the instrument’s own quality metric.

**Figure 2:**
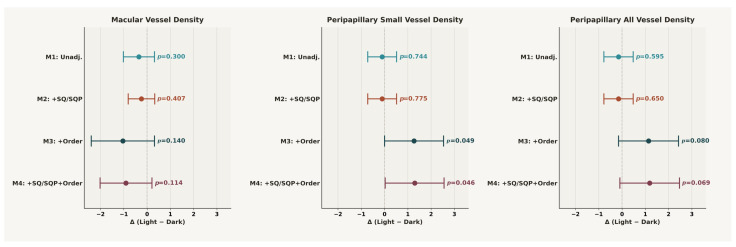
Forest plot of the light-adaptation effect (Δ Light − Dark) on VD across four nested models. Values are from linear mixed-effects models with a participant-level random intercept. Four nested models were fitted per metric: M1 (unadjusted), M2 (+scan quality), M3 (+sequential position 1–12), and M4 (+scan quality + sequential position). Displayed estimates are adjusted for scan quality and measurement order (M4); error bars denote 95% confidence intervals.

Figure 2 presents a forest plot summarizing the light-adaptation effect (Δ Light − Dark) on VD across four nested linear mixed-effects models. For macular VD, the point estimate was negative in all specifications, but none reached statistical significance (M1 unadjusted: Δ = −0.35%, *p* = 0.30; M2 SQ-adjusted: Δ = −0.24%, *p* = 0.40; M3 order-adjusted: Δ = −1.04%, *p* = 0.14; M4 fully adjusted: Δ = −0.91%, *p* = 0.11), and the 95% confidence intervals consistently crossed zero. In contrast, the peripapillary parameters exhibited a marked shift in the estimated effect once measurement order was included as a covariate. For peripapillary small VD, the unadjusted and SQ-adjusted estimates were near zero and non-significant (M1: *p* = 0.74; M2: *p* = 0.77), but the order-adjusted models revealed a significant positive light-adaptation effect (M3: Δ = +1.27%, *p* = 0.049; M4: Δ = +1.30%, *p* = 0.046), with the confidence interval shifting entirely above or near zero. A similar pattern was observed for peripapillary all VD, where the effect became borderline significant after order adjustment (M3: Δ = +1.14%, *p* = 0.080; M4: Δ = +1.19%, *p* = 0.069). These results indicate that the fixed examination sequence masked the true light-adaptation response in the peripapillary region: once the progressive signal decline across the 12-measurement sequence was statistically controlled, a positive adaptation effect emerged for small VD and approached significance for all VD.

**Figure 3:**
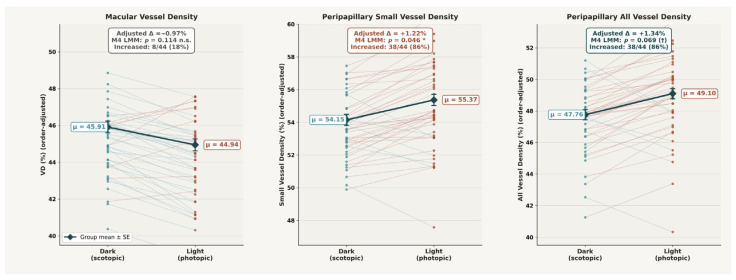
Within-eye dark-to-light change in OCTA VD after adjustment for examination order and scan quality. Each thin line connects one eye’s order-corrected dark and light values (red: increase, blue: decrease); black diamonds show group means ± SE. Values are from linear mixed-effects models with a participant-level random intercept. Displayed estimates are adjusted for scan quality and measurement order (M4); error bars denote 95% confidence intervals. Macular VD showed no significant change (Δ = −0.91%, *p* = 0.11; 18% increased), whereas peripapillary small (Δ = +1.30%, *p* = 0.046; 86% increased) and all VD (Δ = +1.19%, *p* = 0.069; 86% increased) increased under light adaptation, confirming a region-specific peripapillary vasodilatory response.

Examining the OCTA measurements in their sequential examination order (positions 1–12, with positions 1–6 corresponding to dark-adapted and positions 7–12 to light-adapted conditions) revealed distinct trajectories across the three regions. For macular VD, the trend across positions was essentially flat (β = −0.068 per position), consistent with the non-significant order effect observed in the mixed-effects models. In contrast, peripapillary small VD showed a progressive decline of approximately 0.9 percentage points over the full sequence (β = −0.078 per position × 11 position steps ≈ −0.86%), with a comparable trajectory for peripapillary all VD (β = −0.077 per position). This progressive decline—likely attributable to patient fatigue, reduced fixation stability, or tear-film disruption during the approximately 20 min examination session—appears to have masked the underlying light-adaptation response in the unadjusted analyses. Once measurement order was statistically controlled in the M3 and M4 models, a clear positive effect of light adaptation emerged in the peripapillary region (Figure 3), confirming that the region-specific vasodilatory response was obscured by, rather than absent from, the raw sequential data. Participant sex had no significant effect on VD and did not significantly modify the light-adaptation effect in any region when entered as a covariate in the mixed-effects models.

Finally, a cluster-bootstrap resampling procedure confirmed M4 stability (intervals reported here are bootstrap-based and may differ slightly from the parametric model-based CIs in Table 2): for peripapillary small VD, the light-adaptation effect was robust (bootstrap mean β = +1.12; 95% CI: 0.02–2.21; significant in 77.5% of resamples), and the sequential position effect was consistently negative (95% CI: −0.37 to −0.01). For peripapillary all VD, the light-adaptation effect was borderline (95% CI: −0.10 to 2.17; 69.0% significance rate), while for macular VD, the scan quality effect was highly stable (95% CI: 1.26–2.08) but the adaptation effect was not (62.3% significance rate; 95% CI: −2.45 to 0.55). These results support the robustness of the order-unmasked peripapillary finding despite the limited sample size.

## 4. Discussion

The present pilot study demonstrates that OCTA is capable of capturing vascular reactivity associated with light adaptation, consistent with neurovascular coupling responses in the peripapillary region during physiological visual stimulation. An increase in peripapillary small-vessel density was observed under photopic compared to scotopic conditions, indicating that OCTA can detect hemodynamic changes associated with light adaptation. At the same time, no corresponding difference was identified in the macular region. Our peripapillary finding is concordant with Nelis et al. [74], who reported a significant increase in peripapillary vessel density from dark to light conditions in healthy subjects using the same OCTA platform family, supporting the physiological plausibility of an adaptation-related peripapillary response. An important methodological observation of this study is the influence of examination sequence on vessel density measurements. The applied protocol, in which dark-adapted measurements preceded light-adapted measurements, resulted in a systematic decline across successive scans. This pattern suggests that progressive signal degradation or fatigue-related factors may obscure subtle physiological changes, including NVC responses. After accounting for this sequence effect, the light adaptation–related increase in peripapillary vessel density became more evident, reinforcing the ability of OCTA to detect NVC-associated vascular changes. These findings highlight that technical and procedural factors can meaningfully influence OCTA-derived metrics. Importantly, these considerations extend beyond NVC-focused investigations. Both signal quality and examination order should be carefully taken into account in all OCTA studies when interpreting vessel density measurements, as they may introduce systematic bias or mask true biological effects. A related observation about temporal modulation of flicker-induced hyperemia has been reported by Aung et al. [76], who used laser speckle flowgraphy and showed that stimulus duration affected the magnitude and evolution of optic-disk and peripapillary blood-flow responses (with longer 60 s flicker producing larger and more sustained responses than 10 s flicker).

The pronounced dissociation between the peripapillary and macular responses is plausibly explained by distinct anatomical and functional characteristics of the two circulations, including differing metabolic demands, vascular architecture, and autoregulatory mechanisms [11,17]. The macula is characterized by a dense, multilayered capillary network supporting the high metabolic demand of central vision, whereas the peripapillary region is dominated by radial peripapillary capillaries supplying the retinal nerve fiber layer. We offer the following as hypotheses requiring confirmation: (1) the radial peripapillary capillaries are closely coupled to ganglion-cell axonal activity and may therefore respond more strongly to adaptation stimuli, so that peripapillary vessel density changes may more accurately reflect dynamic neurovascular responses; (2) the dense, multilayered, autoregulated macular network may buffer subtle functional changes, rendering macular measurements less responsive to stimuli such as light adaptation; (3) macular VD was analyzed as a composite measure across the full retinal thickness rather than separating individual plexuses, because deep capillary plexus segmentation is less reliable and more susceptible to projection artifacts and noise in standard clinical OCTA, which may dilute a layer-specific signal; and (4) scan quality exerts a stronger influence on macular than on peripapillary measurements, potentially obscuring small macular differences.

Scan quality emerged as a substantial confounding factor influencing macular vessel density quantification by 1.62% per SQ unit. Importantly, a similar magnitude and statistically significant impact of image quality on OCTA-derived vessel density has already been consistently demonstrated across multiple studies in healthy subjects [62], diabetic populations [46,65], and patients with carotid artery stenosis [1,52]. Notably, the influence of scan quality on macular vessel density was comparable to the ~1.3% physiological light-adaptation effect of interest. These results imply that even minor fluctuations in SQ can mask or mimic physiological NVC responses when the true effect size is small, as in the present protocol. Mechanistically, reduced signal strength diminishes sensitivity to small-caliber capillaries, pushing them below the detection threshold and artificially lowering vessel density. Consequently, rigorous image quality control is mandatory for any longitudinal or interventional study: SQ should be tightly matched through inclusion criteria, monitored in real time, and incorporated into the analysis either as a covariate or via correction algorithms that normalize vessel density to a reference SQ, since failure to account for image quality risks misattributing technical artifacts to biological change. Clinically, progressive cataract or other media changes can systematically decrease image quality and thus measured vessel density, potentially mimicking disease progression or NVC impairment [62,63,64,65]. These considerations emphasize that future NVC-oriented OCTA systems should incorporate stronger, more stable illumination, higher signal-to-noise ratio, and optimized acquisition speed, and that both acquisition and post-processing must be explicitly optimized to decouple true physiological NVC signals from image quality-related variability.

The observed 1.3% increase in peripapillary vessel density during light adaptation is consistent with physiological expectations, as increased photopic activity typically induces vasodilation and elevates blood flow through functional hyperemia [7]. A further consideration is that OCTA-derived vessel density quantifies the proportion of perfused area but does not directly measure blood flow velocity or volumetric flow [43]. A reduction in capillary caliber in the macular region, accompanied by stable or even increased flow velocity, may result in lower vessel density values despite preserved or elevated total flow; this mechanism may also contribute to the absence of higher vessel density under illumination in the macula. Previous studies using DVA consistently report 3–6% increases in retinal arteriolar diameter during flicker-light stimulation, reflecting classic hyperemic responses to increased neuronal activity [77,78,79]. This apparent discrepancy in the direction and magnitude of responses likely stems from fundamental differences in measurement principles, sampling, and stimulation paradigms. DVA interrogates larger arterioles and venules and uses high-frequency flicker (typically 12.5 Hz), while the current study probes a composite capillary-level perfusion signal in the macula under natural dark-to-light adaptation using OCTA. DVA also measures vessel diameter directly with high temporal resolution, capturing continuous dynamics, whereas OCTA provides quasi-static snapshots of a flow-derived signal at discrete time points [80]. Thus, DVA and OCTA may be sampling different segments of the vascular tree under different stimulus conditions and capturing distinct yet complementary aspects of NVC. Despite these differences, both modalities detect hemodynamic responses to neuronal activation, collectively supporting the concept that OCTA is sensitive to NVC-related signals, albeit through an indirect surrogate (vessel density) with different temporal and spatial characteristics than DVA.

From a translational perspective, understanding and quantifying NVC is of considerable importance, as NVC disruption is an early hallmark of several neurovascular and neurodegenerative diseases, including diabetic retinopathy, glaucoma, Alzheimer’s disease, and vascular cognitive impairment. Recent functional OCTA work in humans has demonstrated reversed NVC during dark-to-light adaptation as the earliest detectable abnormality preceding clinical diabetic retinopathy [69]. These findings suggest that OCTA-based NVC metrics have the potential to serve as sensitive biomarkers of early neurovascular impairment and treatment response. To the best of our knowledge, this is the first demonstration that OCTA-derived vascular density measurements obtained with a standard, commercially available device can detect region-specific NVC-related hemodynamic alterations during physiological stimulation in humans. Our results extend this concept to a standard clinical OCTA platform under physiological adaptation, showing that an NVC-related signal is detectable but small, vulnerable to technical confounders, and regionally specific to the peripapillary area, which primarily reflects ganglion cell and retinal nerve fiber layer metabolism, in contrast to the macular region, where no significant vessel density change was observed. At the same time, the small effect size and strong dependence on image quality underscore that conventional OCTA platforms, optimized primarily for static structural imaging, are not yet ideal for robust NVC biomarker applications and likely require dedicated hardware and protocol refinements; because the present study was conducted in healthy participants, it establishes feasibility rather than diagnostic performance in any specific disease.

This study has several limitations. Foremost, adaptation condition and measurement order were completely confounded by design—all dark-adapted scans preceded all light-adapted scans—so illumination status and sequential position are inseparable, and no statistical adjustment can fully distinguish a genuine light-adaptation effect from a progressive, order-related decline in signal. Because the primary peripapillary finding emerged only after adjustment for order, it should be interpreted as an association consistent with—but not proof of—a physiological adaptation response, and a non-physiological explanation cannot be excluded. Further limitations include the absence of an independent neurovascular coupling reference (e.g., DVA), a modest sample size (22 participants), a small adjusted effect magnitude (~1.3%) of uncertain clinical significance, and technical variability—particularly scan quality—comparable to the biological signal. These findings should therefore be regarded as preliminary and hypothesis-generating. Confirmation will require larger cohorts using a counterbalanced or randomized acquisition order combined with a same-session DVA reference, alongside a shortened protocol with brief inter-scan rest periods, lubricating artificial tears to stabilize the tear film, and continued statistical adjustment for measurement order.

In light of these observations, future development of OCTA-based NVC biomarkers in humans will likely require systems designed explicitly for functional imaging. Such platforms should integrate higher-power, temporally well-controlled stimulation (e.g., built-in flicker modules or standardized luminance steps), high-speed acquisition with improved motion correction, stable and high-signal-to-noise illumination, and advanced algorithms for image-quality normalization and flow quantification. By increasing the magnitude and reproducibility of the NVC signal, these next-generation functional OCTA systems could improve the separation between healthy and diseased eyes, enable robust longitudinal monitoring, and support the use of NVC metrics as endpoints in clinical trials. Within this framework, the present study can be viewed as a proof-of-concept demonstration that standard OCTA is already capable of detecting physiological, region-specific NVC-related changes in retinal vessel density while simultaneously revealing the limitations that must be addressed before OCTA-derived NVC metrics can be reliably adopted as clinical biomarkers.

## 5. Conclusions

This pilot study demonstrates that OCTA can detect vascular reactivity associated with light adaptation, consistent with NVC during physiological dark-to-light adaptation, evidenced by a significant 1.3% increase in vessel density under photopic versus scotopic conditions. This supports the use of OCTA not only for structural but also for functional assessment of retinal NVC, particularly when imaging protocols are tightly controlled. A key methodological finding is that scan quality significantly influences measured capillary blood flow (vessel density), with a 1.62% change in macular vessel density per SQ unit, underscoring the need to include SQ as a covariate to avoid confounding the neurovascular signal. This highlights that rigorous scan quality standardization and statistical control are essential to avoid misattributing technical variation to biological change, especially in longitudinal or interventional designs. NVC–related changes were confined to small vessel density in the peripapillary region, with no significant effects for macular vessel density, suggesting that peripapillary vessel density is the most sensitive OCTA-derived metric for adaptation-related hemodynamic responses. Although DVA remains superior for real-time characterization of NVC kinetics and amplitude, OCTA’s broad clinical availability, rapid acquisition, and layer-specific imaging make it an attractive complementary modality for functional neurovascular assessment. Given the retina’s role as a window to cerebral microcirculation and neurodegenerative disease, the ability of OCTA to capture NVC responses opens avenues for screening neurovascular dysfunction, monitoring disease progression, and evaluating therapies affecting the CNS. Future studies should validate these findings in larger cohorts using multimodal imaging and longitudinal follow-up in both healthy and diseased populations to optimize OCTA-based neurovascular-coupling protocols and support their standardized clinical and research implementation. Building on this work, we plan to apply this dark-to-light adaptation protocol—with a counterbalanced acquisition order and a parallel DVA reference—in patients with mild cognitive impairment and Alzheimer’s disease, in whom impaired NVC is anticipated.

## Figures and Tables

**Table 1:** Baseline characteristics of the study participants.

Parameter	Mean (SD)	Range
Age (years)	68.3 ± 5.2	50–74
Gender (F/M)	14/8	
Visual Acuity	0.99 ± 0.03	0.8–1.0

**Table 2:** Extended linear mixed-effects models incorporating sequential examination order (position 1–12) as an additional covariate alongside scan quality.

Variable	β Coefficient (%)	95% CI (%)	*p*-Value
Macular Vessel Density			
Light condition (photopic/scotopic)	−0.91	−2.02–0.21	0.11
SQ (scan quality)	1.62	1.23–2.02	<0.001
Sequential position	0.11	−0.05–0.28	0.18
Peripapillary Small Vessel Density			
Light condition (photopic/scotopic)	+1.30	0.03–2.56	0.04
SQP (scan quality)	0.13	−0.34–0.60	0.60
Sequential position	−0.23	−0.43–−0.03	0.02
Peripapillary All Vessel Density			
Light condition (photopic/scotopic)	+1.19	−0.09–2.47	0.06
SQP (scan quality)	0.21	−0.27–0.69	0.39
Sequential position	−0.22	−0.42–−0.02	0.02

CI: confidence interval; SQ: scan quality (macular); SQP: scan quality (peripapillary). All models include the patient as a random intercept. Sequential position ranges from 1 (first measurement, right eye dark-adapted) to 12 (last measurement, left eye light-adapted).

## Data Availability

The data presented in this study are available on request from the corresponding author.

## Abbreviations

The following abbreviations are used in this manuscript:

DVA	Dynamic Vessel Analyzer
OCTA	Optical Coherence Tomography Angiography
NVC	Neurovascular Coupling
VD	Vessel Density
SQ	Scan Quality
SQP	Scan Quality Peripapillary
CI	Confidence Interval
CNS	Central Nervous System
